# Hearing Loss and Neuropsychiatric Symptoms in Persons With Dementia at the End of Life

**DOI:** 10.1093/geroni/igab046.747

**Published:** 2021-12-17

**Authors:** Carrie Nieman, Natalie Regier

**Affiliations:** 1 Johns Hopkins University School of Medicine, Baltimore, Maryland, United States; 2 School of Nursing, Johns Hopkins School of Nursing, Maryland, United States

## Abstract

Access to effective communication is critical to the conversations that occur at end-of-life and represents an unaddressed need within palliative care. These challenges may disproportionately affect persons with dementia (PWD). Hearing loss is one of the most common comorbidities among PWD and is independently associated with neuropsychiatric symptoms. However, relatively little is known about the potential impact of hearing loss on PWD at end-of-life. We examined last month of life (LML) data from 971 proxies of deceased PWD from the National Health and Aging Trends Study (2011-2020). Hearing difficulty was associated with increased anxiety/sadness in PWD, χ2(1)=4.596, p=.032, such that 65.6% of persons with hearing difficulty reported anxiety/sadness in the LML. Binary logistic regression found that hearing difficulty was significantly associated with increased anxiety/sadness (OR=1.40, 95% C.I. 1.00 – 1.80, p < 0.05). Interventions that optimize communication for PWD may be a meaningful approach to improving the end-of-life experience.

